# Finite-Element-Mesh Based Method for Modeling and Optimization of Lattice Structures for Additive Manufacturing

**DOI:** 10.3390/ma11112073

**Published:** 2018-10-23

**Authors:** Wenjiong Chen, Xiaonan Zheng, Shutian Liu

**Affiliations:** State Key Laboratory of Structural Analysis for Industrial Equipment, Dalian University of Technology, Dalian 116024, China; wjchen@dlut.edu.cn (W.C.); 201231127@mail.dlut.edu.cn (X.Z.)

**Keywords:** lattice structures, additive manufacturing, infilled structure finite-element-mesh based method, MIST method

## Abstract

A parameterization modeling method based on finite element mesh to create complex large-scale lattice structures for AM is presented, and a corresponding approach for size optimization of lattice structures is also developed. In the modeling method, meshing technique is employed to obtain the meshes and nodes of lattice structures for a given geometry. Then, a parametric description of lattice unit cells based on the element type, element nodes and their connecting relationships is developed. Once the unit cell design is selected, the initial lattice structure can be assembled by the unit cells in each finite element. Furthermore, modification of lattice structures can be operated by moving mesh nodes and changing cross-sectional areas of bars. The graded and non-uniform lattice structures can be constructed easily based on the proposed modeling method. Moreover, a size optimization algorithm based on moving iso-surface threshold (MIST) method is proposed to optimize lattice structures for enhancing the mechanical performance. To demonstrate the effectiveness of the proposed method, numerical examples and experimental testing are presented, and experimental testing shows 11% improved stiffness of the optimized non-uniform lattice structure than uniform one.

## 1. Introduction

Lattice structure is a kind of high-efficiency cellular material, which attracts the attention of researchers and engineers for their significant potential of lightweight applications and multifunctional design opportunities, such as superior mechanical properties (including energy absorption, strength, and stiffness) [1], heat transfer and thermal protection properties [2]. However, due to the complex geometries, it is difficult for the conventional technique of manufacturing such as extrusion and molding to fabricate lattice structures directly [3,4,5], which restricts their application and development. The majority of available designs are uniform lattice structures composed of periodic unit cells. However, a non-uniform lattice structure with variable unit cells and complex microstructure can achieve significantly better performance relative to the uniform one through optimizing the gradient variation of unit cells and the areas and/or orientations of the bars. Additive manufacturing (AM) is an emerging technique that provides a great flexibility for the fabrication of complex structures, and gives engineers great freedom to fabricate novel lattice structures with complex geometries [6,7,8]. Thus, AM technologies make the manufacture of the non-uniform lattice structures possible [9,10]. To make the most use of design space, a geometric modeling method of lattices is needed to take advantage of the shape complexity capabilities of AM. Moreover, a highly efficient design method is also required to optimize the large-scale lattice structure for enhancing mechanical performances.

The engineering or physiological structures often have complex shapes. The majority of constructive approaches are targeted to design the internal geometry of a structure by filling it with the periodic repetition of regular unit cells [11,12,13]. The advantages of such periodic porous structures consist in their easier modeling and fabrication, as well as in the possibility of predicting their structural properties. Due to such a regularity and periodicity, it is very difficult to exert local control on pore shape, size and distribution, since a minute modification of the unit cell will turn into global changes to the entire structure. More importantly, it is very difficult, if not impossible, to construct lattice structures fitting to complex geometric boundaries. Usually, Boolean operations (e.g., intersection) performed on the acquired model of the structure and the arranged stack of cellular units are required for the generation of lattices with particular external shape. Consequently, the boundary cellular units will be cut to satisfy the structural shape, and these defects in lattice structures have a significant effect on mechanical performance. However, a non-uniform lattice structure with variable unit cells and optimized bar sizes and/or orientations not only can achieve better performance than the uniform one, but also can fit the structural boundary shape. Thus, design of conformal lattice structures in this context of pursuing higher-performance is challenging and becomes a focus of recent studies. One of the most important issues is to develop a method to model parametrically the variation of the unit cells in the space, including the sizes of the unit cells and the areas and/or orientations of the bars.

In fact, lattice structures can be considered as truss structures. Thus, the ideas and principles of the available parameterization and optimization methods for truss structures, such as homogenization based method [14,15] and ground-structure method [16], can be used to construct the modeling and optimization method for non-uniform lattice structures.

The homogenization approach employs a composite material as a basis for defining shape in terms of material density. Periodic microstructures are used as the equivalent homogenized material with the same effective properties. Usually, the design of cellular structure by using homogenization approach is considered as a design process at two scales [17,18,19] (i.e., structural topology design at the macro scale and microstructure design at the micro scale). The method requires that the feature sizes of the microstructure are much smaller than the length scale of the global structure. Therefore, this approach may become inaccurate when feature sizes of the microstructure are comparable to the length scale of the global structure. Moreover, the method based homogenization approach is not suitable for design optimization of large-scale non-uniform lattice structures applied in engineering.

The ground truss approach starts with a ground structure, which is a grid of all elements connecting the nodes in the design space. The optimal truss structure is realized by selecting an optimal substructure from this pre-defined ground structure. Ultimately, the ground-truss approach is a sizing optimization problem, where the cross-sections of ground truss members are the continuous design variables for the optimization. The cross-sections of the bars are sized to support the applied loads on the structure. Dorn et al. [20] proposed the well-known ground structure method in 1964, introducing numerical methods to the field of truss optimization. Based on the ground structure method, many studies are carried out. A review about developments of the ground structure method can be found in [21,22]. Zhou and Rozvany [16] proposed a highly efficient new method (DCOC) for the sizing optimization of large structural systems, greatly improving the scale of the truss optimization. In recent years, intelligent optimization algorithms have been applied to the field of truss optimization, e.g., genetic algorithm [23], particle swarm optimization algorithm [24], simulated annealing algorithm [25] and ant colony optimization [26]. The intelligent optimization algorithm is also not suitable for optimization of large-scale lattice structures because of the huge computation cost.

Based on the basic idea of ground-structure method, in which the optimized truss structure is obtained by properly adjusting and selecting the positions of nodes and cross-section areas of bars from the pre-defined ground structure, we proposed a parameterized modeling and optimization method for non-uniform lattice structures in this paper. Though meshing the defined domain using finite mesh technology, the ground-structure is constructed and modeled parametrically based on the finite element mesh information, and a MIST-based method is developed for optimizing the positions of the nodes and the cross-sectional areas of the bars of lattice-structures. Here, MIST (the Moving Iso-Surface Threshold method) is a new topology method which can avoid explicit sensitivity analysis, and thus yields another advantage of simplification in interfacing with an in-house or commercial finite element analysis program [27,28,29]. Large-scale lattice structures conforming to complex shapes can be quickly constructed via the proposed modeling method, benefiting from the development of finite element mesh generation technology. Then, optimized lattice structures with higher performance can be found by optimizing initial lattice structures with the proposed optimization method. The optimization method has a very small amount of calculation for solving large-scale engineering problems of lattice structures.

The remainder of the paper is organized as follows. Section 2 gives a brief introduction about the modeling method of lattice structures based upon finite element mesh, including the parametric description format, generation process and modification of lattice structures. Section 3 introduces the process of the MIST method. In addition, a size optimization algorithm based on MIST method is proposed to design lattice structures. Numerical examples are given to demonstrate the effectiveness and generality of the proposed method in Section 4. Finally, conclusions are given in Section 5.

## 2. Lattice Structure Configuration

### 2.1. Finite Element Mesh Based Modeling Method

The general idea of the modeling method is as follows: Firstly, meshes of the given geometry are realized through the finite element meshing technique. Then, a parametric description of lattice unit cells based on the element type, element nodes and their connecting relationships is developed. The bars of lattice unit cell are established by connecting nodes with the connecting relationships in element. Finally, the lattice structures are generated by assembling the lattice unit cell in each element. The detail process of modeling method is given in this section.

**Figure 1:**
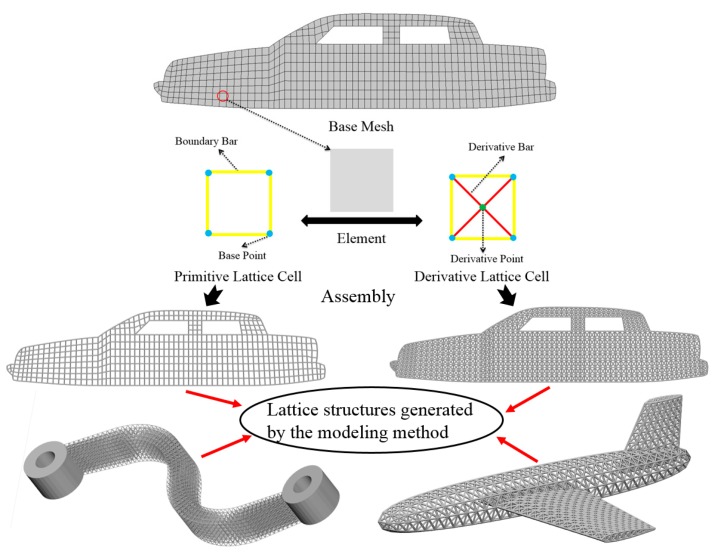
Procedure of the modeling method and some lattice examples.

For convenience, we introduce some definitions as follows:

1. Base mesh: Base mesh is the initial finite element mesh, which is obtained by meshing the given geometry.
2. Base points: Base points are the nodes of the initial finite element mesh.
3. Boundary bars: Boundary bars are the bar on element edge. The end points of boundary bars are base points.
4. Derivative points: Derivative points are the extra nodes introduced to construct the additional bars. The positions of the derivative points are interpolated by the coordinates of base points.
5. Derivative bars: Derivative bars are the bars made by connecting base points and derivative points.
6. Primitive lattice cells: Primitive lattice cells are the unit cells constructed by boundary bars, as shown in Figure 1.
7. Derivative lattice cells: Derivative lattice cells are the unit cell constructed by boundary bars and derivative bars, as shown in Figure 1.

**Figure 2:**
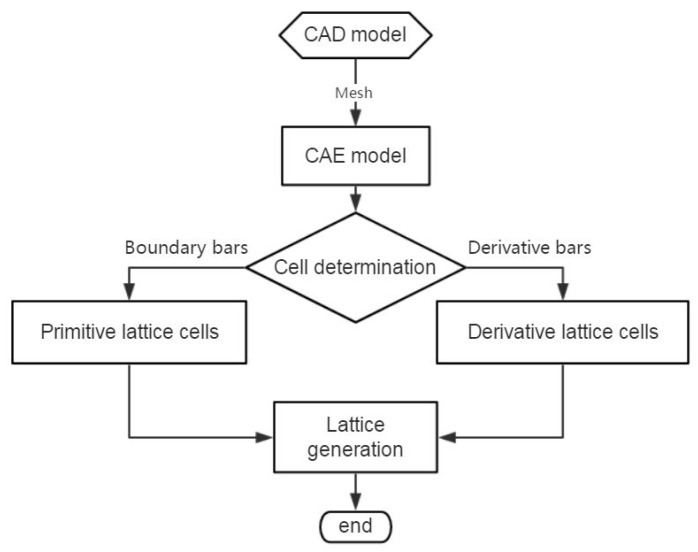
Flowchart of the lattice structure modeling method based upon finite element mesh.

The modeling method is summarized into steps, as shown in the flowchart of Figure 2. The whole procedure of modeling method for larger-scale conformal truss structures based upon finite element is outlined as follows.

Step 1: Finite Element Mesh Generation

For the first step, the given geometry needs to be meshed to get base mesh. Base mesh can provide the initial nodes and elements information of the geometry. It is a process of converting a CAD model to a CAE model. Nowadays, commercial CAE software can provide simple and efficient finite element preprocessor, so Step 1 can be easily implemented by commercial finite element software without extra programming.

Step 2: Determination of the Lattice Unit-Cell

After meshing the given geometries, it is needed to determine the lattice unit-cell models. This paper provides two types of lattice unit-cell models. The first type of unit cell is called primitive lattice cell which are made up of boundary bars. The boundary bars are formed by connecting nodes (base points) of the mesh along the edge of the element. Each type of element corresponds to a certain primitive lattice cell. The second type of cell is called derivative lattice cell which are made up of boundary bars and derivative bars. Derivative points are the extra nodes introduced to construct derivative bars. The positions of the derivative points are interpolated by the coordinates of base points. Then, the derivative bars are formed by connecting the base points of the element and derivative points. For one type of element, many different derivative lattice cells can be obtained by different derivative points. For example, Table 1 shows the element types, primitive lattice cells, derivative lattice cells and the corresponding interpolation formats. In Table 1, only one simple derivative lattice cell with one derivative point is given for each type of element.

Step 3: Lattice Structure Generation

**Figure 3:**
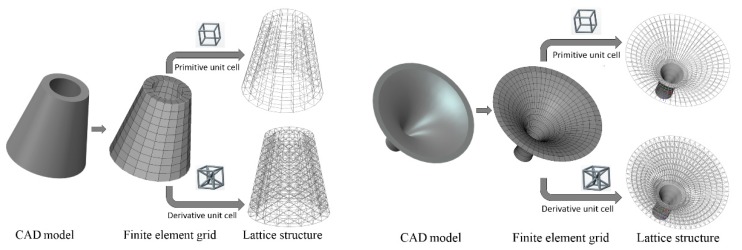
Illustrations of lattice generation for given geometries.

Initial node and element information of the given geometry can be obtained from Step 1, and Step 2 can provide the lattice unit-cell model. The last step is assembly of lattice structure (i.e., infilling the whole geometry with lattice unit-cell model). Figure 3 shows illustrations of lattice structure generation for two given geometries.

### 2.2. Modification

**Figure 4:**
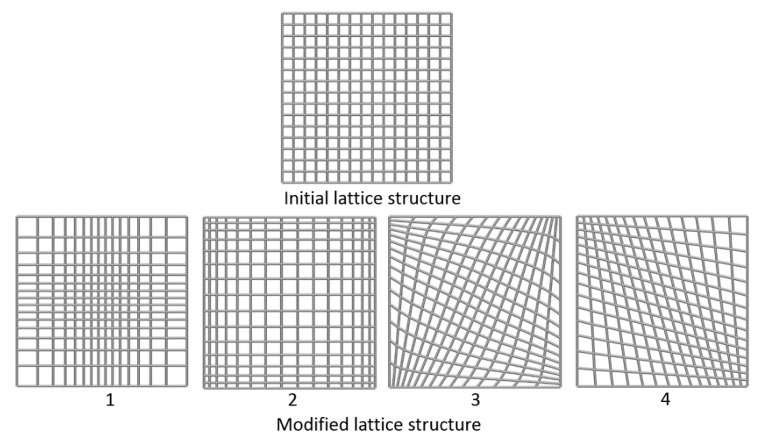
Modification of lattice structures by moving mesh nodes.

**Figure 5:**
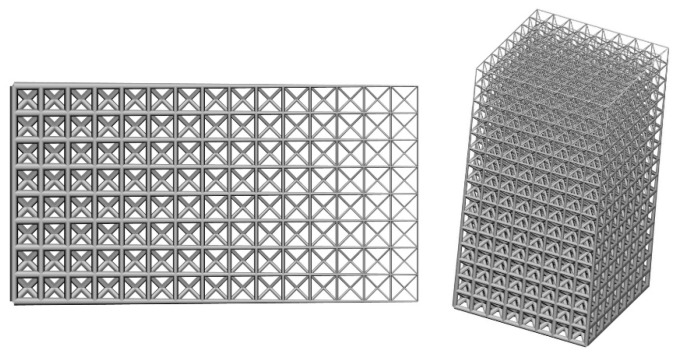
Cuboid lattice structure with linear gradient cross-sectional areas of the bars.

The lattice structures generated by the modeling method are adjustable. The first way to get modified lattice structures is to move mesh nodes of initial lattice structures. For example, by moving the nodes to the stress concentration area, lattice structures with local reinforcement properties can be constructed and the stress in the corresponding area will decrease. Figure 4 shows an example of modification of lattice structures by moving mesh nodes. The second way to get modified lattice structures is to modify the cross-sectional areas of the bars. By changing the cross-sectional areas of the bars, lattice unit-cell model with different volume fractions can be constructed. The non-homogeneous distribution can be achieved by infilling the geometry with different volume fractions unit-cell models. Figure 5 shows a cuboid lattice structure with linear gradient distribution of volume fraction. By the modification of initial lattice structures, non-uniform lattice structures with the desired properties can be constructed easily.

## 3. Problem Statement and Formulation

### 3.1. A Brief Overview of MIST

**Figure 6:**
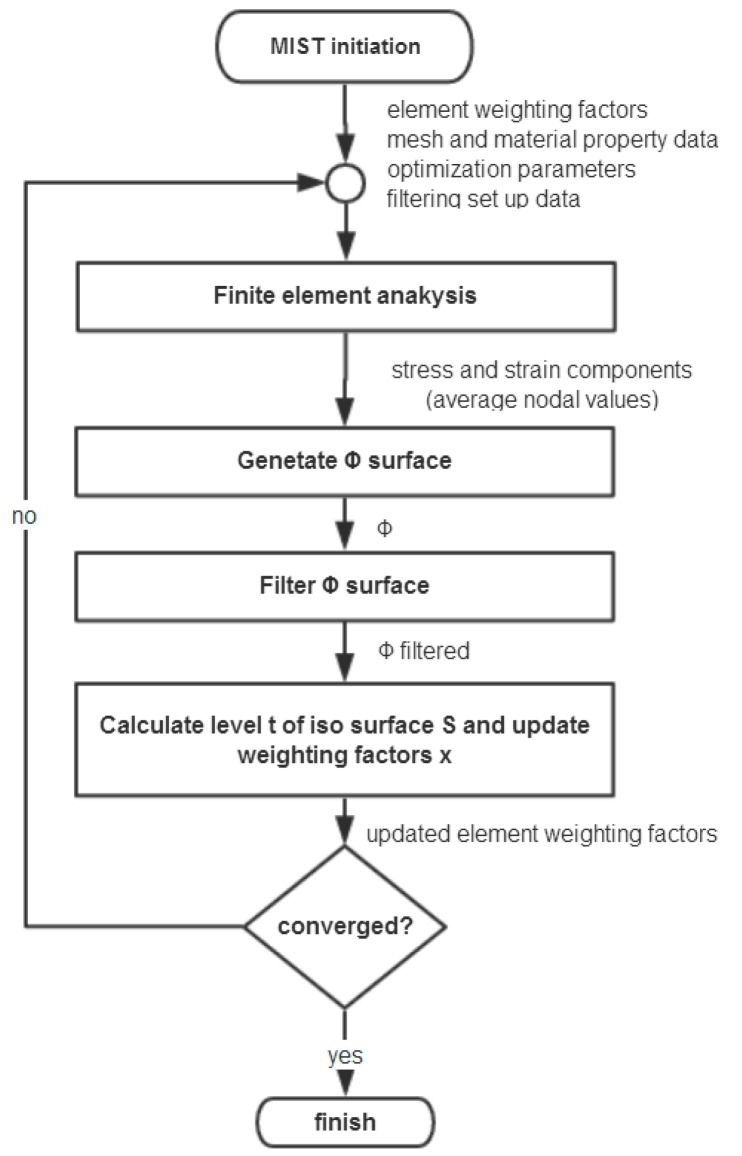
Flowchart of the MIST topology optimization method.

This paper uses a novel method referred as MIST to optimize the cross-sectional areas of the bars of lattice structure. MIST method is a new topology optimization method used to solve topology optimizations, such as shown in Equation (1). The method is proposed by Tong and Vasista in 2014, and the process flowchart of MIST method is presented in Figure 6. The method aims to find the design variable values *x* and *t* to minimize a certain structure objective function, such as the total strain energy. As the basic principle of MIST method, a physical response function Φ (stresses, strains and their appropriate combinations) should be written in an integral form over the design domain. An iso-surface *S* intersects the Φ function and the contour formed by the intersection becomes the structural boundary. The level or threshold value t of the iso-surface depends on the volume constraint and is calculated. Weighting factors are used on the elements to represent the material distribution: void and solid element are represented by weighting factors of 0 and 1, respectively. In the element weighting factor update routine, the element with Φ above the iso-surface at all nodes move towards solid material (weighting factors tend 1), and those elements with Φ below the iso-surface at all nodes move towards void material (weighting factors tend 0).

(1) $$\begin{array}{ll} \mathrm{find} & x,t\\ \min & J(x,t)\\ s.t\text{:} & g_{r}(x,t)=0\\ & g_{s}(x,t)\leq 0\\ & x_{l}\leq x\leq x_{u} \end{array}$$

The iso-surface level S is calculated using an iterative bisection method and element weighting factors are updated. The detail steps are given as follows:

Step 1: Determine $t_{\max_{0}}=\max(\Phi )$ and $t_{\min_{0}}=\min(\Phi )$ for a known Φ.

Step 2: Calculate iso-surface level: $t_{k}=\frac{t_{\max_{k}}+t_{\min_{k}}}{2}$.

Step 3: Calculate $\Phi -t_{k}$ at all nodes; set $\Phi -t_{k}$ to 0.01 and −0.01 at solid and void non-design nodes, respectively; and update $x_{i_{k}}$ for all elements based on area ratio.

Step 4: If $\left|V_{k}-V_{cons}\right|<\zeta$, terminate iteration; otherwise

Case (a) if $V_{k}>V_{cons}\to \left\{ \begin{array}{l} t_{\min_{k+1}}=t_{k}\\ t_{\max_{k+1}}=t_{\max_{k}} \end{array}\right.$

or

Case (b) if $V_{k}<V_{cons}\to \left\{ \begin{array}{l} t_{\min_{k+1}}=t_{\min_{k}}\\ t_{\max_{k+1}}=t_{k} \end{array}\right.$

where ζ is the bisection method tolerance on material constraint. *V_k_* is the total volume of the structure at *k*th iteration step. *V_cons_* is the volume constraint.

**Figure 7:**
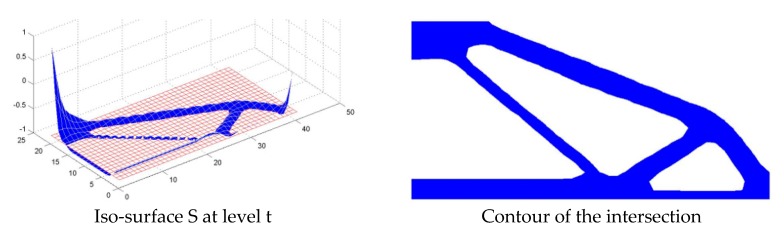
Illustration of a response surface, an iso-surface S and contour of the intersection.

For Step 3, $x_{i_{k}}$ is calculated for each element based on the values of $\Phi -t_{k}$ at the element nodes. If $\Phi -t_{k}$ is positive at all element nodes, then $x_{i_{k}}$ moves towards 1. If $\Phi -t_{k}$ is negative at all element nodes, then $x_{i_{k}}$ moves towards 0. If the values of $\Phi -t_{k}$ are positive at some node(s) and negative at some node(s), then $x_{i_{k}}$ is based on the ratio of projected positive area to total element area. A response surface, an iso-surface and contour of the intersection for a half simply supported beam problem are shown in Figure 7.

### 3.2. Size Optimization Algorithm Based on MIST Method

In this paper, we use the MIST method to optimize the lattice structure, choosing strain energy expression of the bar element as Φ function and cross-sectional areas of the bars as variable values. Upper and lower bounds of the cross-sectional areas need to be defined in advance. Usually, we choose the smallest size limitation of a 3D printer as the lower bound of the cross-sectional area. As for upper bound, we choose five times of the initial bar length as the upper bound of the bar diameter. Bar element only has two nodes so there is a little difference when calculating the variable value *t*. When updating the areas of the bars, if the value of $\Phi -t_{k}$ is positive at one node and negative at the other node, the cross-sectional area of the bar remains unchanged.

Hence, we can formulate the design optimization of lattice structures problem for maximizing equivalent stiffness as follows:

(2) $$\begin{array}{ll} \mathrm{find} & A=\left\{A_{i}\right\},\;i=1,\ldots ,N\\ \min & f(A)=\sum_{i=1}^{N}\Phi_{i}\\ s.t. & F=KU\\ & \sum_{i=1}^{N}A_{i}L_{i}<V_{cons}\\ & 0<A_{l}\leq A_{i}\leq A_{u},\;i=1,\ldots ,N \end{array}$$

where *A_i_* is the cross-sectional area of bar number *i*, f(A) is objective function, Φ is the response function for MIST, *F* is load vector, *K* is overall stiffness matrix, *U* is displacement vector, *A_l_* is the lower bound of the cross-sectional area, *A_u_* is the upper bound of the cross-sectional area, and $V_{cons}$ is the volume constraint.

In this paper, the linear elastic constitutive model shown as Equation (3) is adopted into the optimization algorithm without considering the plasticity and yielding effects.

(3) $$\sigma =\bar{E}\cdot \varepsilon$$

The initial cross-sectional areas of each bar are the same and depend on the volume constraint. As mentioned, an appropriate selection of the response function Φ is crucial; in this paper, the Φ function used was the strain energy of the bar as given in Equation (4).

(4) $$\Phi_{i}=\frac{F_{i}^{2}\cdot L_{i}}{2\cdot E\cdot A_{i}}$$

where *L_i_* is the length of bar number *i*, *F_i_* is the axial force of bar number *i*, *E* is Young’s modulus, and *A_i_* is the cross-sectional area of bar number *i*.

## 4. Numerical Results

To demonstrate the effectiveness of the proposed method, several numerical examples are presented in this section. The sizes and loads for all examples are chosen to be dimensionless. Young’s modulus and Poisson’s ratio of the material are selected as *E* = 2.0 × 10^5^ and *v* = 0.3, respectively. For the convenience of description, the design variable of cross section *A* is replaced by the circular radius *r*. The relationship between *r* and *A* is $A_{i}=\pi \cdot {r_{i}}^{2};A_{l}=\pi \cdot {r_{l}}^{2};A_{u}=\pi \cdot {r_{u}}^{2}$.

### 4.1. Numerical Examples for 2-Dimensional Structures

(A) A 2D cantilever beam

**Figure 8:**
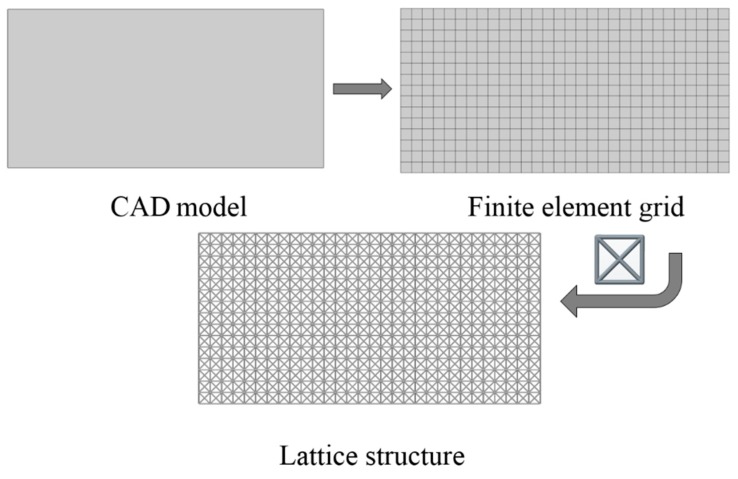
Illustrations of lattice structure generation for 2D cantilever beam.

**Figure 9:**
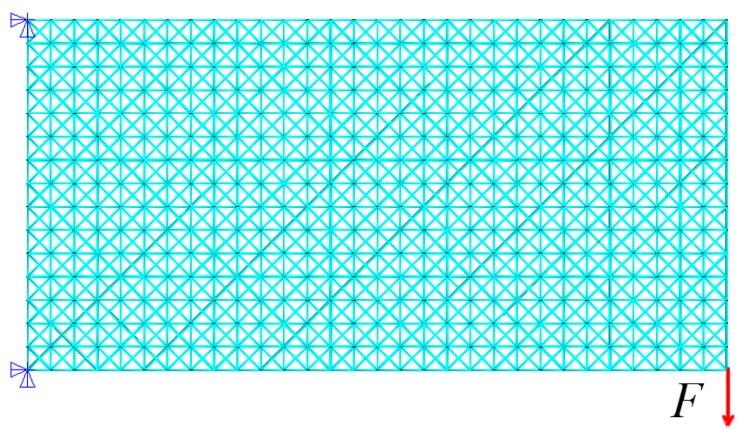
The initial lattice structure, loading and boundary conditions for cantilever beam.

**Figure 10:**
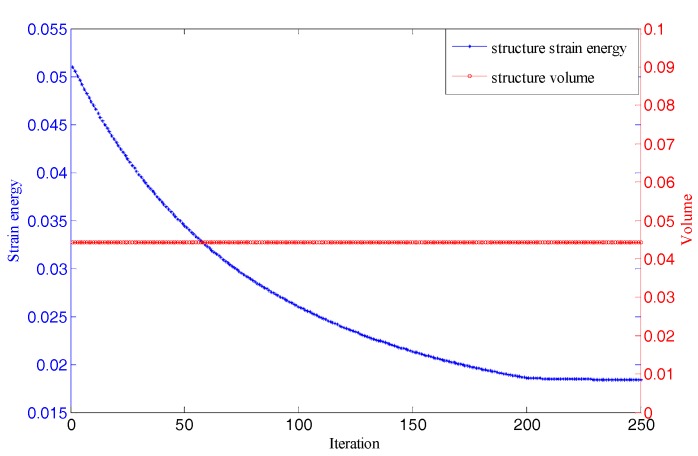
Iteration histories of the objective and volume for cantilever beam.

**Figure 11:**
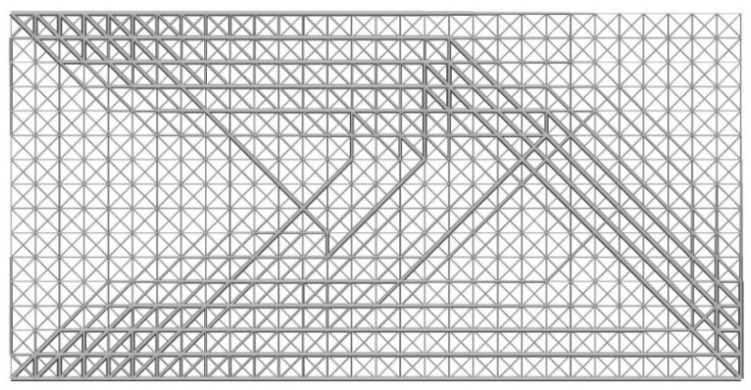
Final optimized structure of the cantilever beam.

As the first test case, a 2D cantilever beam is considered. Figure 8 shows the process of initial lattice structure generation for 2D cantilever beam. Figure 9 shows its finite element model, initial lattice structure, boundary conditions and load. The overall size of cantilever structure is 6 × 3 (dividing into 30 × 15 unit cells). The structure is fixed at the top-left and bottom-left corner point and loaded at bottom-right corner (F = 1000). The initial radius of bar is 0.01, lower bound of the radius is 0.005 and upper bound is 0.02. Figure 10 plots the iteration histories of the compliance of the cantilever structure and volume fraction of the structure. The iteration process is stable, and the objective function and the structure converge to their final solutions after 200 iterations. The compliance of the structure is decreased by 63.9% and the volume fraction is still unchanged. Figure 11 shows the final optimized structure of the 2D cantilever beam. The result is considered reasonable and the pattern layout is similar to the result from topology optimization of continuum structures.

(B) A hollow round with a circular support

**Figure 12:**
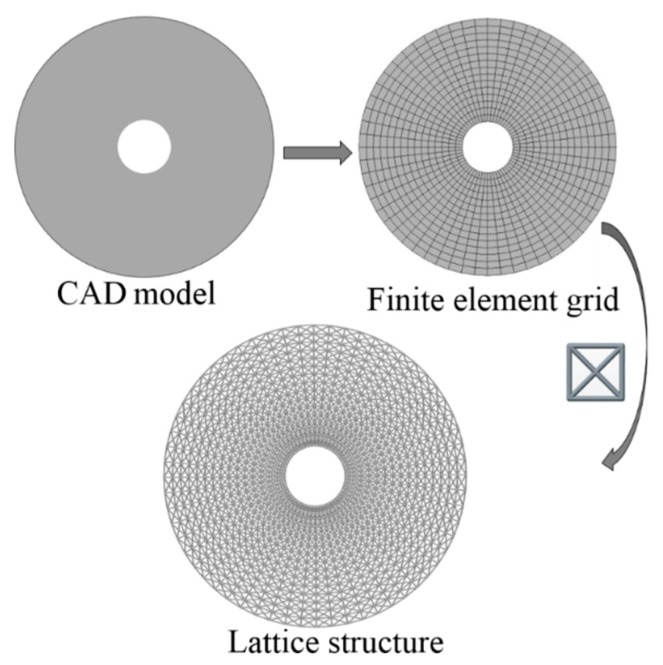
Illustrations of lattice structure generation for hollow round.

**Figure 13:**
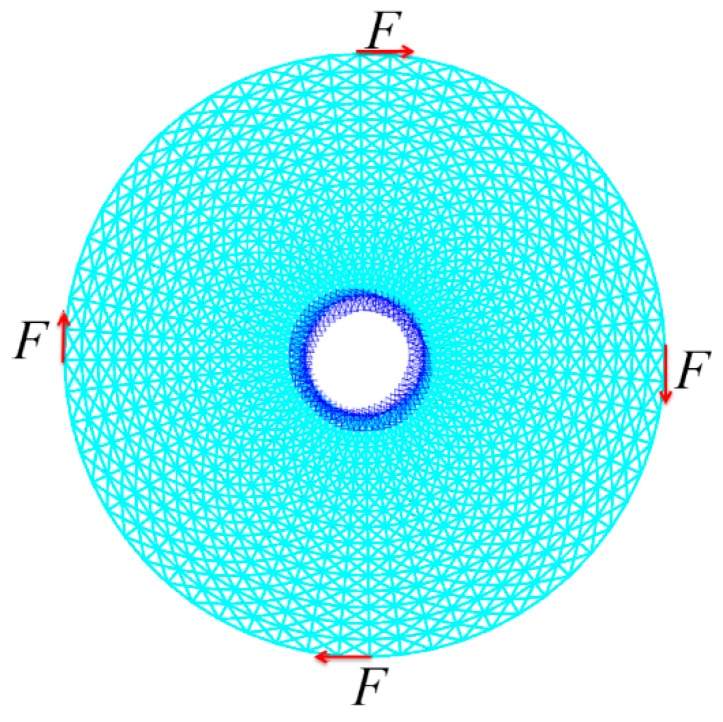
The initial lattice structure, loading and boundary conditions for hollow round.

**Figure 14:**
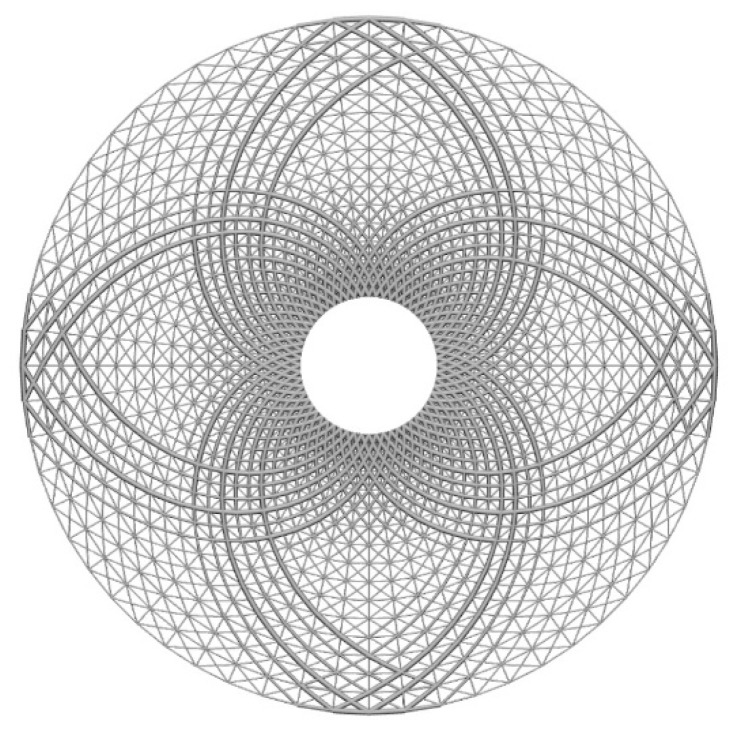
Final optimized structure of the hollow round.

The second example is a hollow round with inside diameter 1 and outside diameter 5. The process of initial lattice structure generation is the same as the last example, and Figure 12 shows the illustrations of lattice structure generation for the hollow round. Figure 13 shows its finite element model, initial lattice structure, boundary conditions and loads. There are 15 unit cells in the radial direction and 60 unit cells in the circumferential directions, the inner boundary points are fixed and tangential loads are applied on the outer boundary (F = 1000). The initial design variable radius is 0.02, lower bound of the radius is 0.01 and upper bound of the radius is 0.04. Figure 14 shows the final optimized structure of the hollow round.

### 4.2. Numerical Examples for Three-Dimensional Structures

(A) A hollow round platform

**Figure 15:**
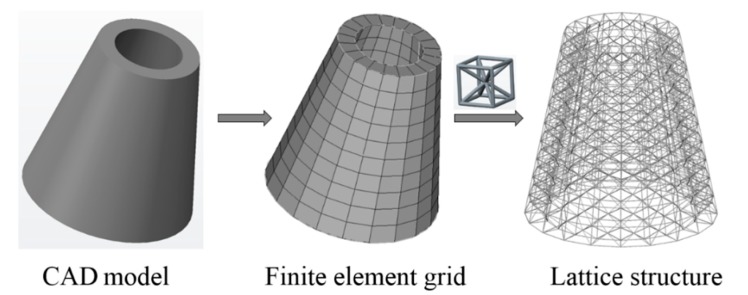
Illustrations of lattice structure generation for hollow round platform.

**Figure 16:**
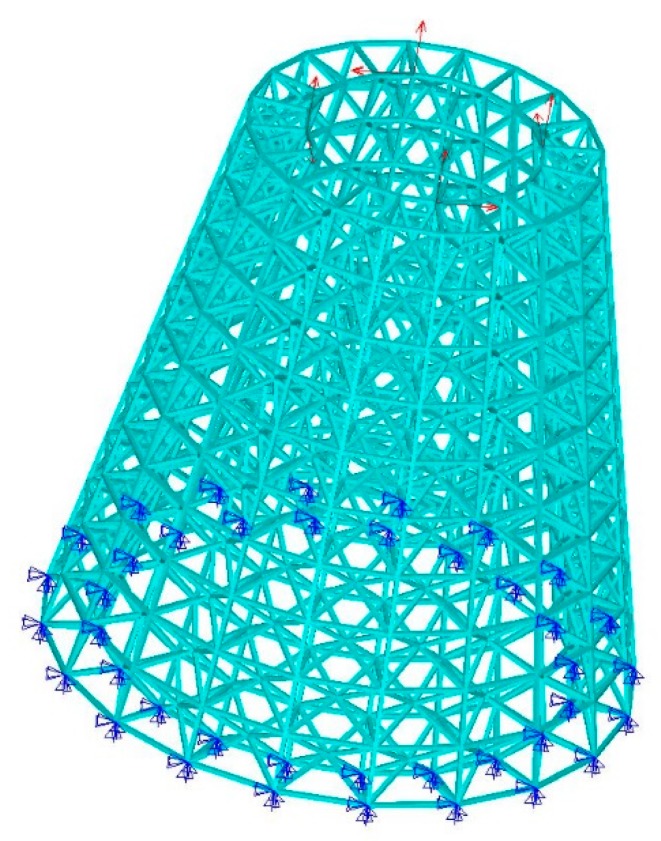
Boundary conditions and loading for hollow round platform.

**Figure 17:**
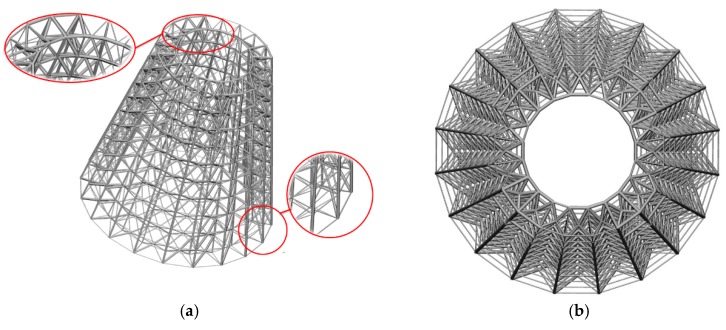
Final optimized structure of the hollow round platform: (**a**) three-dimensional stereogram; and (**b**) vertical view.

**Figure 18:**
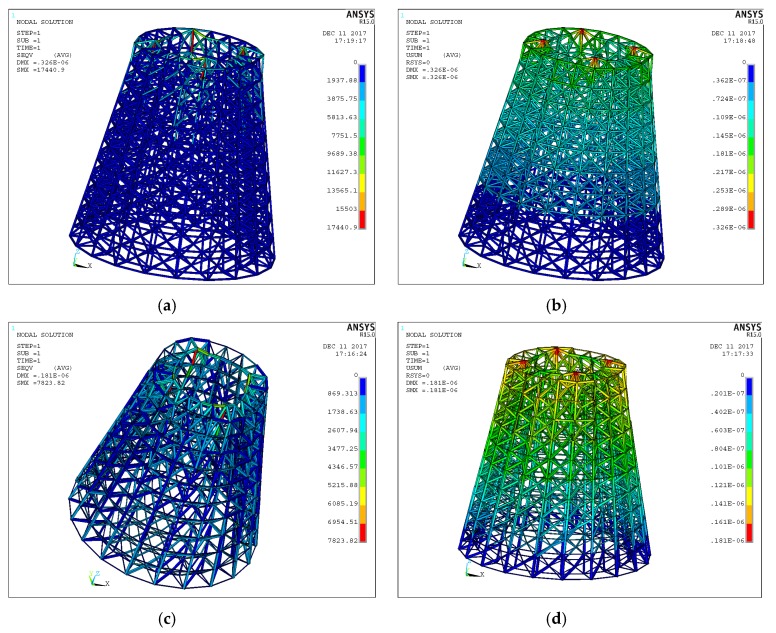
Analysis results: (**a**) von Mises stress for initial structure; (**b**) displacement for initial structure; (**c**) von Mises stress for optimized hollow round platform; and (**d**) displacement for optimized hollow round platform.

For a hollow round platform, the process of initial lattice structure generation for the structure is shown in Figure 15. Figure 16 shows the finite element model, loading and boundary conditions of the hollow round platform. The bottom of the hollow round platform is fixed, tangential and tensile loads are applied on four top-inside points of the hollow round platform. The initial radius of bar is 0.04, lower bound of the radius is 0.02 and upper bound of the radius is 0.06. Figure 17 shows the final optimized structure. Figure 17a shows the local details of the optimized structure, and we can clearly see the different thickness of the bar. In this case, we compare initial structure with optimized structure in stiffness and strength by numerical simulation. Figure 18 shows the results of the numerical simulation. After the optimization, the maximum Mises stress of the structure is down by 55.14% and the maximum displacement is down by 44.48%. Apparently, our method can find better lattice structures both in stiffness and strength.

(B) A three-dimensional horn structure

**Figure 19:**
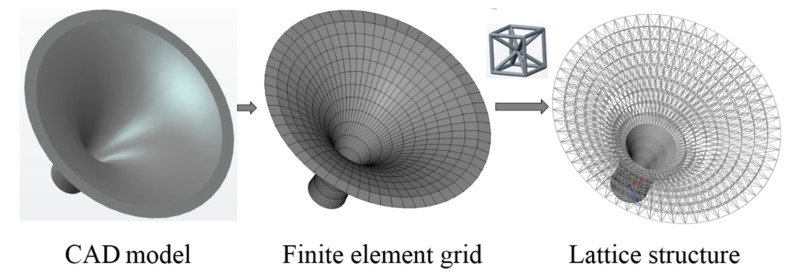
Illustrations of lattice structure generation for horn structure.

**Figure 20:**
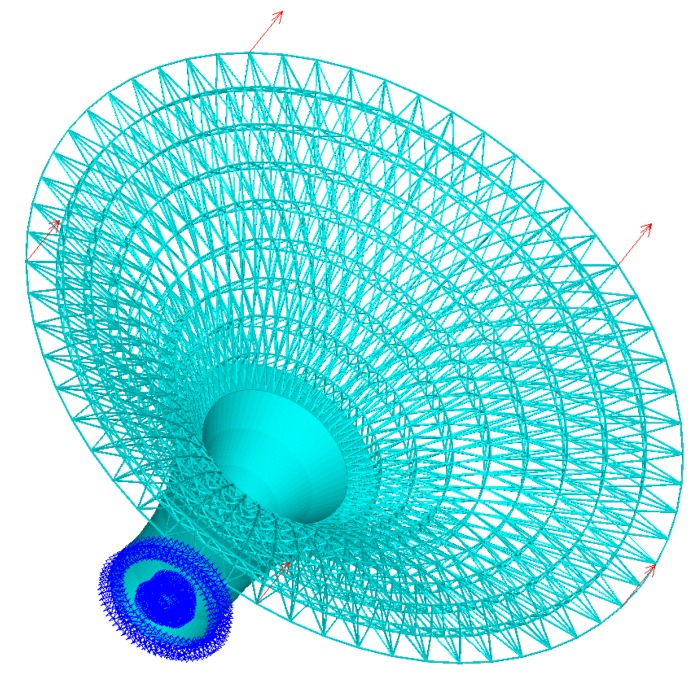
Boundary conditions and loading for horn structure.

**Figure 21:**
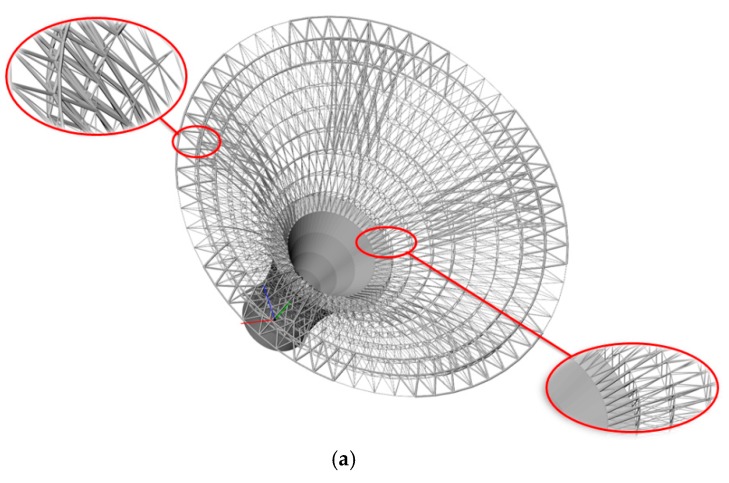

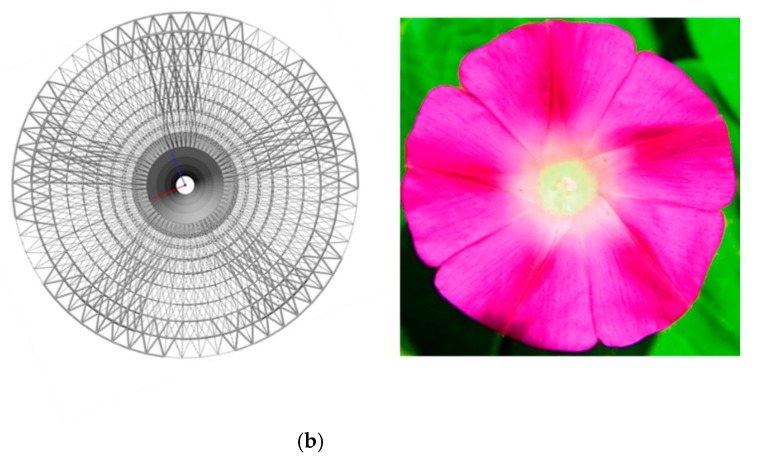
Final optimized structure of the horn structure: (**a**) three-dimensional stereogram; and (**b**) vertical view.

The second three-dimensional example is a horn structure. The model consists of a lattice structure and a solid structure. The same with the hollow round platform, we give the initial lattice structure directly. Illustrations of lattice generation for the structure are shown in Figure 19. Figure 20 shows the finite element model, boundary conditions and loading of the horn structure. The bottom of the horn is fixed and the structure is subjected to five vertical point loads on the top of the structure. The initial radius is 0.02, lower bound of the radius is 0.01 and upper bound of the radius is 0.04. Figure 21a is the three-dimensional stereogram of the optimized horn and it shows the local details of the optimized structure. Figure 21b shows the vertical view of the final optimized structure. We compared the optimized structure with that of the flower shown in Figure 21b, and we found that they are similar in terms of functionality and appearance. Apparently, our method is also applicable to the mixed model of solid structures and lattice structures.

## 5. Experimental Validation

### 5.1. Models for the Test

**Figure 22:**
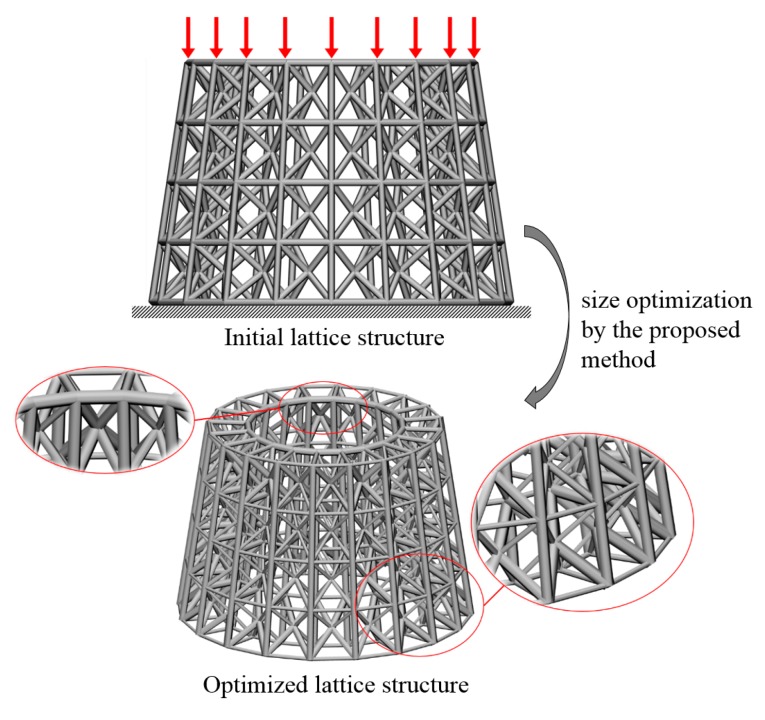
Boundary conditions and result of the optimization problem.

**Figure 23:**
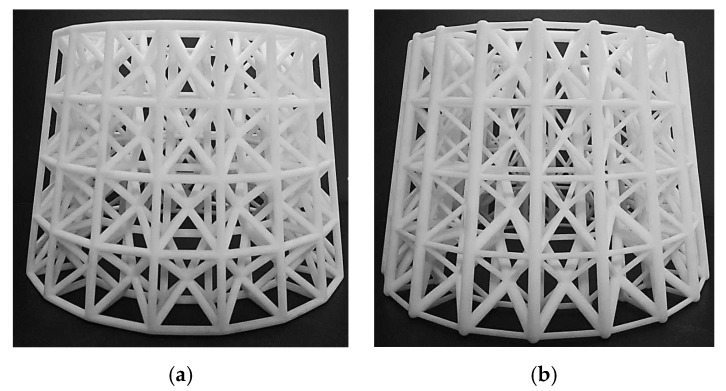
3D printed samples of: (**a**) initial lattice hollow round platform; and (**b**) optimized lattice hollow round platform.

To further verify the effectiveness of the proposed method, an experimental validation is carried out for a 3D example. The structure similar to the first 3D numerical example is selected as the experimental object. Some changes are made to the model to make the experiment more convenient to carry out. Due to the limitation of 3D printer’s processing precision, the lower bound of the radius of the rods cannot be too small. Considering the size of the experimental platform, the overall size of the model also needs to be adjusted. Therefore, the stiffness increase of the experimental model after optimization cannot reach the value in the numerical study. In addition, surface texture produced by AM also has a very important impact on structural performance [30,31]. Figure 22 shows the boundary conditions and result of the optimization problem. Both the initial and optimized lattice hollow round platform in Figure 23 are additively manufactured with SLA (Stereo Lithography Apparatus) printing technology. Droplets of photo-sensitive liquid resin are selectively deposited onto a substrate through a movable nozzle, and simultaneously solidified through ultraviolet light.

### 5.2. Mechanical Test and Result

**Figure 24:**
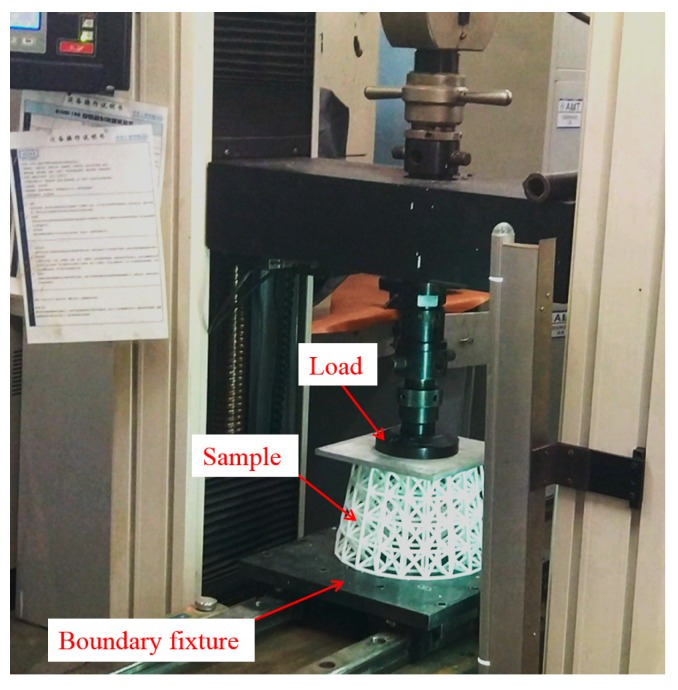
Experimental setup.

**Figure 25:**
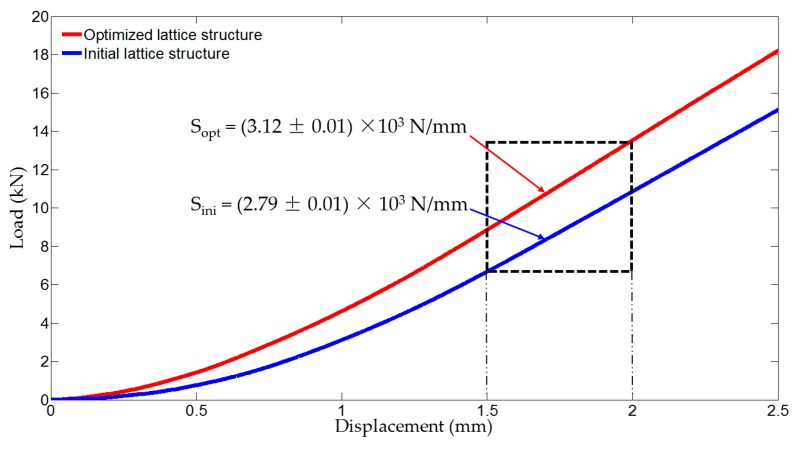
Load–displacement curves for experimental verifications.

Experimental validation is carried out for both initial and optimized lattice structures shown in Figure 23 for mechanical test. The experiments are conducted on a WDW-100 electromechanical universal testing machine (Kexin, Changchun, China) with a 100.0 kN load cell, according to the experimental setup shown in Figure 24. All of the specimens are loaded by dropping the top bracket downwards at a speed of 0.5 mm/min, which is to simulate the quasi-static condition. Each test is stopped at maximum displacement of 2.50 mm. Figure 25 shows the measured load–displacement responses of the specimens. Since the specimens are only tested for stiffness validation within the linear elastic region as well as under the geometrical linearity condition for small displacements, the curves within the displacement range between 1.5 and 2.0 mm are used to evaluate the stiffness of the samples. The global stiffness of the specimens is evaluated by linear regression, with resulting values of S_ini_ = (2.79 ± 0.01) × 10^3^ N/mm and S_opt_ = (3.12 ± 0.01) × 10^3^ N/mm for the initial and optimized hollow round platform, respectively. The measured stiffness values clearly demonstrate the superior stiffness of the optimized structure over the initial structure, with a significant improved global stiffness of 11.83%. Here, experiments are conducted only for comparing stiffness between initial and optimized lattice structures, which is in general a standard requirement for most engineering applications.

## 6. Conclusions

This paper presents a new approach to model and design lattice structure for additive manufacturing. Lattice structures conforming to complex shapes can be constructed by the modeling method. In the modeling method, the meshing techniques are employed to obtain the discrete nodes of lattice structures for a given geometry. Then, a parametric description of lattice unit cells filled in meshes is developed to assist the user in assembling the architecture of lattice structures. Once a unit cell design is selected and sized, the initial lattice structure can be assembled by the unit cell in each finite element. Furthermore, modified lattice structures can be obtained by adjusting the initial lattice structures. By moving grid nodes, graded and non-uniform lattice structures can be constructed. By changing the cross-sectional areas of the bars, the lattice unit-cell model with different volume fractions can be constructed.

The modeling approach based upon finite element method can be used for generating large-scale lattice structure for any kind of geometry. Moreover, the MIST method provides a useful method for designing large-scale lattice structures. However, further investigation is needed to evaluate its efficiency and effectiveness compared with other algorithms. Several numerical examples are provided to illustrate the validity of the proposed method. After optimization, the maximum Mises stress of the hollow round platform example is decreased by 55.14% and the maximum displacement is decreased by 44.48%. The experimental results also demonstrate the benefits of performing non-uniform design by using the proposed method. The measured stiffness values clearly demonstrate the superior stiffness of the optimized structure over the initial structure, with a significantly improved global stiffness of 11.83%. In addition, an important conclusion can be made that the optimized non-uniform lattice structure is able to provide a higher stiffness than the uniform design. However, fabricating 3D optimized lattice structures may encounter issues such as removal of support structures, surface roughness and surface texture, which could significantly influence the mechanical performance of the samples. This situation will be considered in our next work.

## Figures and Tables

**Table 1:** Illustrations of elements, primitive cells, derivative cells and interpolation formats.

Element Type	Triangle	Quadrangle	Tetrahedron	Hexahedron
Element and nodes	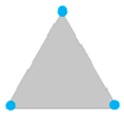	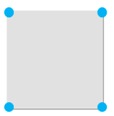	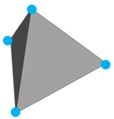	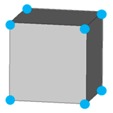
Primitive cell	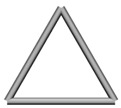	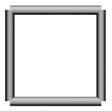	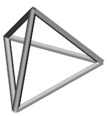	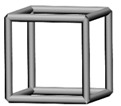
Derivative cell	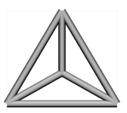	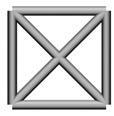	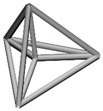	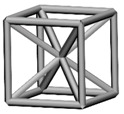
Number of derivative points	1	1	1	1
Interpolation format	$\begin{array}{l} x_{Der}=\frac{1}{3}\sum_{i=1}^{3}x_{i}\\ y_{Der}=\frac{1}{3}\sum_{i=1}^{3}y_{i}\\ z_{Der}=\frac{1}{3}\sum_{i=1}^{3}z_{i} \end{array}$	$\begin{array}{l} x_{Der}=\frac{1}{4}\sum_{i=1}^{4}x_{i}\\ y_{Der}=\frac{1}{4}\sum_{i=1}^{4}y_{i}\\ z_{Der}=\frac{1}{4}\sum_{i=1}^{4}z_{i} \end{array}$	$\begin{array}{l} x_{Der}=\frac{1}{4}\sum_{i=1}^{4}x_{i}\\ y_{Der}=\frac{1}{4}\sum_{i=1}^{4}y_{i}\\ z_{Der}=\frac{1}{4}\sum_{i=1}^{4}z_{i} \end{array}$	$\begin{array}{l} x_{Der}=\frac{1}{8}\sum_{i=1}^{8}x_{i}\\ y_{Der}=\frac{1}{8}\sum_{i=1}^{8}y_{i}\\ z_{Der}=\frac{1}{8}\sum_{i=1}^{8}z_{i} \end{array}$
$x_{Der},\;y_{Der},\;z_{Der}$ are the Cartesian co-ordinates of derivative points $x_{i},\;y_{i},\;z_{i}$ are the Cartesian co-ordinates of node number *i*				
